# Awareness, knowledge and belief regarding bitter leaf use: A cross-sectional study in Nigeria

**DOI:** 10.1371/journal.pone.0322364

**Published:** 2025-06-03

**Authors:** Obi Peter Adigwe, Godspower Onavbavba, Ofure Omoarelojie

**Affiliations:** National Institute for Pharmaceutical Research and Development, Abuja, Federal Capital Territory, Nigeria.; Kampala International University - Western Campus, UGANDA

## Abstract

**Background:**

*Vernonia amygdalina,* also known as bitter leaf, is a plant that is widespread in Nigeria. Bitter leaf plant has several medicinal properties, and the plant is also widely used due to its various gastronomic applications. This study aimed to assess awareness, knowledge, and beliefs regarding bitter leaf use.

**Methods:**

A cross-sectional study was undertaken in Nigeria. Paper-based questionnaires were administered to participants, and the data were analysed using Statistical Package for Social Sciences.

**Results:**

Of the 500 questionnaires that were administered, a total of 401 copies were completed and returned, resulting in a response rate of 80.2%. About two-thirds (65%) of the study participants were females, whilst 35% were males. Almost all the participants (98%) had heard about bitter leaf, the total mean score for knowledge of bitter leaf use amongst the respondents was 4.80 ± 2.14 (Range 0–9). Using the Bloom cut off, only about 11.7% of the population had good knowledge and 27.2% had moderate knowledge regarding bitter leaf. However, more than three-quarters of the sample (79.6%) linked bitter leaf to its glucose lowering properties, towards optimal maintenance of blood sugar levels. The respondents’ sources of information on bitter leaf use were mainly from relatives (88%) and social media (19.9%). Statistically significant findings revealed stronger belief amongst females regarding the employment of bitter leaf as a weight loss intervention (*p *= 0.042).

**Conclusion:**

Although most participants were familiar with the bitter leaf plant, only a few of them had adequate knowledge of its properties. Given its widespread use, a comprehensive understanding is imperative to prevent misuse. Findings from this study indicate that most people rely on informal sources for information about the plant, potentially leading to misconceptions regarding proper use. Consequently, evidence-based public education is needed to promote safe consumption and fully harness the plant’s nutritional and medicinal benefits.

## Background

Herbal medicines are naturally occurring plant materials utilised for treating and managing ailments [1]. The history of plants’ medicinal use dates back to the beginning of human existence, and evidence suggests a significant role in the management of diseases [2]. Due to perceived effectiveness, availability and cost friendliness, about 80% of the world population use one form of herbal medicine or the other for their primary health care needs [3].

Over the years, several plants have been analysed for their nutritional constituents. In 2016, the Royal Botanic Gardens Kew estimated the medicinal properties of about 17,810 plant species from approximately 30,000 plants [4]. One of such important plant was *Vernonia amygdalina,* which is commonly referred to as bitter leaf due to its taste. The plant is widely grown in the tropical regions of the world. Bitter leaf plant is widespread in Nigeria, where its leaves and roots are often employed to prepare infusions, juices, cataplasms and powders for various ailments [5]. Additionally, having been successfully domesticated across different parts of the country, the plant is widely used for culinary activities due to its nutritional benefits [6]. Beyond Nigeria, *Vernonia amygdalina* is also widely utilised in other regions. Indigenous communities in the southern region of Ghana predominantly use the young, succulent leaves for a variety of ailments [7,8]. In Ethiopia, the leaves serve as hops in the preparation of ‘tela’ beer, a traditional alcoholic beverage [9]. Additionally, evidence from Tanzania suggests that wild chimpanzees self-medicate with the plant against parasites [10].

*Vernonia amygdalina* has several pharmacological properties, including antimicrobial, anti-malarial, antithrombotic, antioxidant, anti-diabetic, and laxative activities [5,11]. Several studies attribute these activities to the bioactive compounds extracted from its leaves and roots [12]. These bioactive compounds include flavonoids, alkaloids, and tannins [13]. Moreover, cold water extracts of *Vernonia amygdalina* have been reported to exhibit diverse therapeutic effects. For instance, they have demonstrated the ability to suppress cancer [14–16]. Additionally, these extracts possess anti-inflammatory properties [17], Adedapo *et al.* [18] demonstrated that 100 and 200 mg/kg doses of acetone extract significantly reduced carrageenan and histamine-induced edema. Also, the analgesic effects observed were comparable to those produced by indomethacin, the study’s reference drug. These extracts also exhibit hypoglycaemic activity [19–21] and confer neuroprotective benefits [22]. Preclinical studies demonstrated that the ethanolic extract of *V. amygdalina* effectively inhibits Plasmodium berghei [23]. In a clinical trial involving patients aged 12 years and older with uncomplicated malaria, an infusion of fresh *V. amygdalina* leaves produced an adequate clinical response in 67% of cases at day 14; however, only 32% achieved complete parasite clearance, with 71% experiencing recrudescence [24]. Notably, the treatment was well tolerated with no significant side effects.

Given these well-documented benefits, it is important to consider other relevant elements which influence the consumption of *Vernonia amygdalina*. It is well known that beliefs, habits, social factors, and availability are critical factors, influencing plant consumption behaviour [25]. However, previous studies have focused primarily on its pharmacological properties, while little is known about public perceptions and consumption behaviours. To address this gap, the study aimed at assessing awareness, knowledge, and belief regarding the use of *Vernonia amygdalina* in Nigeria. It is envisaged that the findings from this study would provide valuable insights to identify knowledge gaps and inform future research on its pharmacological properties and potential community-based utilisation.

## Methods

The study adopted a cross-sectional design and was undertaken in Nigeria. Data were collected from 30^th^ May 2022 – 28^th^ July 2022. The data collection instrument (See supplementary file) was designed in English language following an extensive review of literature [26–30]. The questionnaire validation process was carried out by an expert panel comprising faculty members that are engaged in teaching and research activities in this field. A draft version of the questionnaire items was reviewed independently by each member of the panel and changes were suggested. The revision process continued until a consensus was achieved. The questions on the instrument were structured to gain insights on knowledge, awareness and belief regarding bitter leaf use. A pilot testing of the questionnaire was undertaken with a sample of 20 participants, who were randomly selected to participate in the study. The feedback received revealed that further modification was not required.

Using the Epi Info software version 7, a sample size of 384 was calculated for a population of approximately 200 million people in Nigeria at 95% confidence level, 5% margin of error, and 50% response distribution. To accommodate for non-response, the sample size was rounded up to 500. The participants were selected using a stratified multistage sampling across the 6 geopolitical zones in the country. One state was randomly selected from each of the zones. In each state, a number of participants were recruited using convenience sampling technique [31]. Strategic locations which include motor parks, markets, worship centres, and corporate offices were visited for data collection.

Ethical approval was received from the National Institute for Pharmaceutical Research and Development Health Research Ethics Committee prior to data collection. Written informed consent was obtained from the participants before administering the questionnaires to them. Inclusion criteria for participants in the study were Nigerian citizenry, willingness to participate, and attainment of the legal age for consent (18 years and above). Participants who did not meet these criteria were excluded from the study. Any term not understood by the participants was explained to them while completing the questionnaire.

Data collected were coded and analysed using the Statistical Package for Social Sciences (SPSS), version 25. Questions assessing knowledge were answered as *true, false, or I don’t know*. A correct answer was assigned 1 point, and an incorrect answer as well as unanswered questions were assigned 0 point. The knowledge score ranged from 0 to 9. Participants’ overall knowledge was categorised using Bloom’s cut-off point as good if the score was between 80% and 100% (7.2–9), moderate if the score was between 60 and 79% (5.4–7.11), and poor if the score was less than 60% (<5.4). Other variables were summarised using frequency and percentage. Inferential statistical analysis was performed using Independent t-test, chi square test and analysis of variance (ANOVA). A *p-*value 0.05 or less was considered statistically significant.

## Results

### Demography

A total of 500 questionnaires were administered, out of which 401 copies were completed and returned, thereby giving a response rate of 80.2%. The mean age of the participants was 34 ± 12.5, two thirds of the participants (65%) were females, and slightly above a quarter of the respondents were educated up to tertiary education level. Other relevant details about socio-demographic characteristics are presented in Table 1.

**Table 1:** Socio-demographic characteristics of the respondents.

Variable	Frequency (%)
Gender	
Male	140 (35.0)
Female	261 (65.0)
Occupation	
Unemployed	62 (15.5)
Student	31 (7.7)
Self employed	69 (17.2)
Employed in public sector	146 (36.4)
Employed in private sector	52 (13.0)
Retired	1 (0.2)
Others	16 (4.0)
Missing data	24 (6.0)
Level of Education	
Primary	4 (1.0)
Secondary	3 (0.7)
Tertiary	264 (65.8)
Postgraduate	112 (27.9)
Missing data	18 (4.5)
Monthly Income	
<30,000	108 (26.9)
31,000-60,000	181 (45.1)
61,000-90,000	33 (8.2)
91,000-120,000	15 (3.7)
Above 120,000	22 (5.5)
Missing data	42 (10.5)

### Awareness

Considering the wide distribution of bitter leaf in Nigeria, findings from this study revealed that almost all the study participants (98%) had heard about the plant. Furthermore, the findings showed that about 93.7% of the study participants consume bitter leaf, and 99.7% of those that use the plant indicated that they utilised the leafy part.

**Fig 1:**
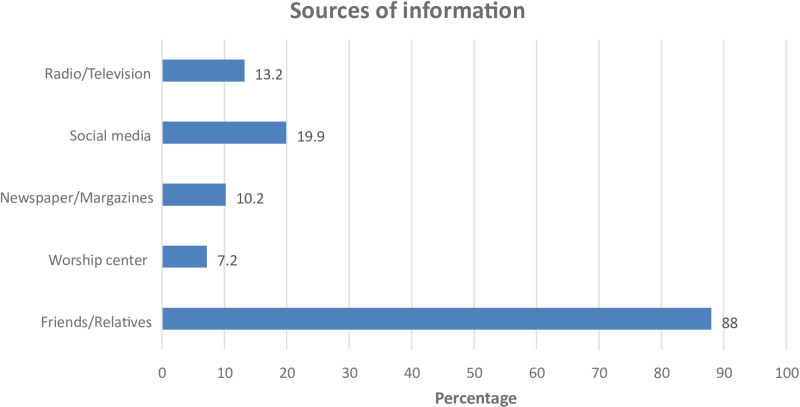
Sources of information about bitter leaf.

Findings presented in Fig 1 show that the respondents obtained information regarding bitter leaf from a variety of sources. The common source of information about the plant was relatives and friends.

Also, a fifth of the participants (19.9%) indicated social media as their source of information regarding bitter leaf. The least indicated source of information for the study cohort, was worship centre.

### Knowledge

The overall mean score for knowledge of bitter leaf use amongst the respondents was 4.80 ± 2.14. Using Bloom’s cut-off, it was revealed that a quarter (27.2%) of the participants had moderate knowledge about bitter leaf, and only about a tenth (11.7%) of the respondents reported good knowledge regarding the plant. Two-thirds of the study participants (61.1%) had poor knowledge of the use of bitter leaf.

Findings also indicated that only 40.1% of the respondents were knowledgeable about the anti-malarial property of bitter leaf. Two-thirds of the respondents (64.6%) were of the view that bitter leaf consumption is an alternative way of incorporating antioxidants. Additionally, more than three-quarters (79.6%) of the participants indicated that bitter leaf consumption can help maintain normal blood sugar level. Other relevant details relating to knowledge regarding bitter leaf use are presented in Table 2.

**Table 2:** Knowledge regarding bitter leaf use among respondents.

SN	Statement	Correct response (%)
1	Bitter leaf contains some important biological active ingredients.	343 (85.5)
2	Bitter leaf consumption could be an alternative way of incorporating antioxidants.	259 (64.6)
3	Regular intake of bitter leaf can help to minimise the risk of stroke	189 (47.1)
4	Incorporation of bitter leaf in daily diet can help maintain normal blood sugar level.	319 (79.6)
5	Bitter leaf is relatively not toxic and safe for consumption.	301 (75.1)
6	Excessive consumption of bitter leaf can increase the risk of anemia.	82 (20.4)
7	Bitter leaf has anti-malaria properties.	161 (40.1)
8	Regular intake of bitter leaf can help minimise the risk of heart attack.	158 (39.4)
9	Excessive intake of bitter leaf can result in stomach upset.	113 (28.2)

### Belief

This study revealed that 70.3% of the respondents believed that it was good to consume bitter leaf after an excessive intake of sugar, whilst close to a third of the respondents (29%) had a positive belief that bitter leaf can reduce the risk of cancer. Furthermore, a significant proportion of the respondents (39.8%) indicated that bitter leaf consumption aids in weight loss. Other relevant details are presented in Table 3.

**Table 3:** Belief regarding bitter leaf use.

SN	Statement	Agree (%)	Disagree (%)	I don’t know (%)
1	Medicinal property of bitter leaf is associated with its bitter taste.	217 (57.0)	71 (18.6)	93 (24.4)
2	It is important to consume bitter leaf following excessive intake of sugar.	267 (70.3)	29 (7.6)	84 (22.1)
3	The nutritional benefits of bitter leaf are highest from the first wash.	260 (68.4)	34 (8.9)	86 (22.6)
4	The nutritional benefit of bitter leaf is reduced when overcooked.	260 (68.6)	46 (12.1)	73 (19.3)
5.	Multiple wash can reduce the nutritional content of bitter leaf.	265 (69.7)	47 (12.4)	68 (17.9)
6	Bitter leaf has adverse effects on individuals.	123 (32.7)	102 (27.1)	150 (40.2)
7	Bitter leaf may contain some toxic minerals.	93 (24.7)	115 (30.5)	169 (44.8)
8	Bitter leaf aids in weight loss.	151 (39.8)	56 (14.8)	172 (45.4)
9	Bitter leaf is widely used due to its nutritional benefits.	319 (86.4)	14 (3.8)	36 (9.8)
10	Eating the leaf raw is the best and most effective way of getting all the nutrients.	175 (45.9)	84 (22.0)	122 (32.0)
11	Lactating mothers can consume bitter leaf to increase breast milk production.	87 (22.8)	57 (15.0)	237 (62.2)
12	Bitter leaf consumption reduces the risk of cancer	109 (29.0)	31 (8.2)	236 (62.8)

### Association between socio-demographic characteristics and knowledge regarding bitter leaf use

Inferential statistical analyses undertaken revealed that gender, age, marital status and level of education of the study participants had no influence on knowledge regarding bitter leaf use as *p *> 0.05 for all the variables. Further details are presented in Table 4.

**Table 4:** Association of demography with knowledge.

Variables	Category	Mean ± SD	Test of significance (*p*)
Gender			t = -0.070 (0.271)
Male		4.78 ± 2.235	
Female		4.80 ± 2.096	
Age			F = 1.540 (0.204)
≤30	a	4.78 ± 2.118	
31-40	b	4.96 ± 2.181	
41-50	c	6.43 ± 1.272	
51-60	d	4.00 ± 1.414	
Level of education			F = 0.111 (0.954)
Primary	a	5.25 ± 1.258	
Secondary	b	4.33 ± 3.786	
Tertiary	c	4.78 ± 2.060	
Post graduate	d	4.79 ± 2.251	

Note: F = analysis of variance (ANOVA); t = Independent t-test.

### Association between socio-demographic characteristics and belief regarding bitter leaf use

A chi square test was undertaken to determine the association between socio-demographic data with the item “bitter leaf aids in weight loss”. Findings revealed that more females were of the view that bitter leaf plant can help reduce weight, and this was statistically significant (*p *= 0.042). Further information relating to this is presented in Table 5.

**Table 5:** Cross-tabulation of socio-demographic characteristics with the belief that bitter leaf aids in weight loss.

Demography	Agree	Disagree	I don’t know	X^2^	*p-value*
Gender				6.318	0.042
Male	50 (40)	27 (20.8)	51 (39.2)		
Female	98 (39.7)	29 (11.7)	120 (48.6)		
Education				5.617	0.467
Primary	2 (50)	1 (25)	1 (25)		
Secondary	2 (100)	0 (0.0)	0 (0.0)		
Tertiary	101 (40.4)	35 (14)	144 (45.6)		
Postgraduate	37 (34.6)	20 (18.7)	50 (46.7)		

## Discussion

A number of new insights emerged regarding knowledge, awareness and beliefs with respect to the utilisation of bitter leaf. Findings from this study revealed that almost all the participants had heard about bitter leaf plant. This was expected due to the widespread cultivation and use of the plant in Nigeria [32]. In this study, the majority of the respondents indicated that they obtained information about bitter leaf from friends and relatives. This may be attributed to high consumption of the plant in different households. The increase in awareness of the use of plants as alternative medicines has necessitated the need for adequate information on the use of medicinal plants [33,34]. Findings from this study revealed that bitter leaf was frequently used by the participants, with a significant proportion of the sample consuming the leaves either as juice or as condiments in soup preparation. These findings validate a previous study that reported regular use of the plant due to its health-related and medicinal benefits in this setting [35].

Despite the high level of awareness and consumption of bitter leaf, the majority of the study participants seemed to have poor knowledge about bitter leaf, suggesting the need for public enlightenment in this area. This is critical, given the fact that the plant is widely consumed in the study setting. This intervention will also mitigate against the incidence of toxicity, as more than two-thirds of the participants were unaware that despite the health and medicinal benefits associated with the plant [36–38], excessive consumption of it can also be harmful [39].

Interestingly, more females had the belief that bitter leaf consumption could aid weight loss. This could be attributed to the rising prevalence of obesity amongst women [40]. The search for effective therapies for weight reduction underpinned by the models identified by this study, may have contributed to their knowledge in this area. In a study carried out by Atangwho *et al.* [41], the anti-obesity effect of *Vernonia amygdalina* was assessed in diet-induced obese rats. Supplementation with *V. amygdalina* led to a noticeable reduction in body weight gain and total body fat. Also, two-thirds of the study participants indicated correctly that bitter leaf has antioxidant properties, suggesting some level of awareness in this area. Previous studies had reported phytochemicals associated with the plant, including flavonoids, saponins, alkaloids, and steroids [42–44]. These phytochemicals are natural bioactive substances known for their anti-oxidative potential and health benefits. However, only slightly above one-third of the sample were aware that bitter leaf was associated with significant antimalarial properties. This appears to be surprising considering the endemic nature of malaria in this part of the world. It has been demonstrated that *V*. *amygdalina* possesses antimalarial properties similar to that of chloroquine [45].

Close to three-quarters of the study participants were of the view that consuming bitter leaf can help in controlling blood glucose level for persons with hyperglycaemia. This suggests that the respondents were knowledgeable about the effect of the plant on blood sugar, as a number of studies had demonstrated the glucose lowering effect of bitter leaf extract [19–21]. Also, two-thirds of the study participants were of the opinion that the nutritional benefits of bitter leaf can reduce when it is over cooked. This finding is in line with extant literature, as it has been established that overcooking of vegetables can result to substantial losses of important components [46].

A quarter of the participants in this study felt that bitter leaf might contain some toxic minerals, possibly because bitter leaf harvested from soil along heavy traffic routes contains significantly higher levels of heavy metals compared to those from other locations [47]. This accumulation occurs as vegetables absorb metals from contaminated soil. Also, whilst about a third of the participants believed that bitter leaf consumption reduces the risk of cancer, a strong majority of the participants had indicated that the plant was widely utilised due to its nutritional benefits. A number of studies had been reported to support anti-cancer activities of the plant [48–52].

## Conclusion

This study has provided new insights into knowledge, awareness and beliefs towards the utilization of bitter leaf plant. Findings revealed that almost all the participants in this study had heard about the plant, however, their knowledge about *V. amygdalina* was poor. Also, almost all the respondents indicated that they consume bitter leaf, and a strong majority of them were of the opinion that the plant was widely utilised due to its nutritional value.

This study has revealed that the significance of bitter leaf cultivation in Nigeria cannot be overemphasised, especially since almost all the respondents indicated that they use the plant. Building on the emergent evidence from this study, the nutritional and medicinal benefits of bitter leaf can be better harnessed, especially following scientific validation. This intervention is particularly useful for improving nutrition, as well as in the management and prevention of indicated diseases, which is a critical public health importance.

Despite the high level of awareness, findings indicate that the majority of the participants rely on informal sources of information, underscoring the need for evidence-based public education to ensure safe consumption. Given the widespread utilization of bitter leaf, especially for both culinary and medicinal purposes, adequate knowledge about its properties is critical, necessitating relevant public awareness campaigns. This intervention is invaluable in preventing inappropriate use, as well as to ensure that citizens derive optimal benefits. Further research can build on emergent findings from this study to catalyse more impactful policy and practice interventions.

## Supporting information

S1 File Questionnaire. (DOCX)

S2 File Dataset. (SAV)

S3 File Consent form. (DOCX)
