# Supplementary material for: Awareness, knowledge and belief regarding bitter leaf use: A cross-sectional study in Nigeria
Source: PLoS One. 2025 Jun 3;20(6):e0322364. doi: 10.1371/journal.pone.0322364 (PMC12132952; doi:10.1371/journal.pone.0322364)
Supplement: S1 File — (DOCX) [file pone.0322364.s001.docx]

**NATIONAL INSTITUTE FOR PHARMACEUTICAL RESEARCH AND DEVELOPMENT, ABUJA**

**Awareness, Knowledge and Belief Regarding Bitter Leaf Use: A Cross-sectional Study in Nigeria**

Bitter leaf (*Vernonia amygdalina*) is mainly consumed for its nutritional value and its role in the maintenance of health. This study aims at assessing knowledge, belief, attitudes, and practices regarding bitter leaf use among persons residing in Federal Capital Territory. Please fill the questionnaire by ticking (✓) the most appropriate option(s). Your responses will be anonymized and treated confidentially.

**Section A: Demography**

1. **Gender**

| Male | Female |
| --- | --- |

1. **Age (Years)……………………….**
2. **Occupation**

| Unemployed | Student | Self-employed | Employed in public sector | Employed in private sector | Retired | Others, please specify …. |
| --- | --- | --- | --- | --- | --- | --- |

1. **Level of education**

| Primary school | Secondary school | Tertiary education | Postgraduate |
| --- | --- | --- | --- |

1. **Monthly Income (₦)**

| ≤ 30,000 | 31,000 -60,000 | 61,000 –90,000 | 91,000 – 120,000 | Above 120,000 |
| --- | --- | --- | --- | --- |

**Section B: Awareness**

1. Have you heard of bitter leaf?

a) Yes

b) No

1. If yes to question 1 above, what is your sources of information (please tick as many as applicable)
2. Relatives
3. Worship centers
4. Newspaper/Magazines
5. Social media
6. Radio/Television
7. Do you consume bitter leaf?
8. Yes
9. No
10. What part of the plant do you use?
11. Leaves
12. Stem
13. Root

**Section C: Knowledge**

| **S/N** | **Statement** | **True** | **False** | **I don’t know** |
| --- | --- | --- | --- | --- |
| 1. | Bitter leaf contains some important biological active ingredients |  |  |  |
| 2. | Bitter leaf consumption could be an alternative way of incorporating antioxidants |  |  |  |
| 3. | Regular intake of bitter leaf helps to minimize the risk of stroke |  |  |  |
| 4. | Incorporation of bitter leaf in our daily diet could help to maintain normal blood sugar level |  |  |  |
| 5. | The bitter leaf plant is relatively not toxic and safe for consumption |  |  |  |
| 6. | Excessive consumption of bitter leaf might increase the risk of anemia |  |  |  |
| 7. | Bitter leaf has a potent anti-malaria property |  |  |  |
| 8. | Regular intake of bitter leaf helps to minimize the risk of heart attack |  |  |  |
| 9. | Excessive intake of bitter leaf can result in stomach upset |  |  |  |

**Section D: Belief**

| **S/N** | **Statement** | **Agree** | **Disagree** | **I don’t know** |
| --- | --- | --- | --- | --- |
| 1. | Medicinal property of bitter leaf is tied to its bitter taste. |  |  |  |
| 2. | Bitter leaf is good following excessive intake of sugar |  |  |  |
| 3. | The nutritional benefits of bitter leaf are highest from the first washed water |  |  |  |
| 4. | The nutritional benefit of bitter leaf when overcooked is reduced |  |  |  |
| 5. | Multiple wash can reduce the nutrient contents of bitter leaf |  |  |  |
| 6. | Bitter leaf has adverse effects on individuals |  |  |  |
| 7. | Bitter leaf may contain some toxic minerals |  |  |  |
| 8. | Bitter leaf aids in weight loss |  |  |  |
| 9. | People use bitter leaf due to it nutritional benefits |  |  |  |
| 10. | Eating the leaf raw is the best and most effective way of getting all the medicinal benefits of the plant. |  |  |  |
| 11. | Lactating mothers consume bitter leaf to increase breast milk production |  |  |  |
| 12. | Bitter leaf consumption reduces the risk of cancer |  |  |  |

**Thank you for taking your time to complete this questionnaire.**
