# Supplementary material for: Awareness, knowledge and belief regarding bitter leaf use: A cross-sectional study in Nigeria
Source: PLoS One. 2025 Jun 3;20(6):e0322364. doi: 10.1371/journal.pone.0322364 (PMC12132952; doi:10.1371/journal.pone.0322364)
Supplement: S3 File — (DOCX) [file pone.0322364.s003.docx]

IRB Research approval number: ####

This approval will elapse on: dd/mm/yyyy

**Title of the research:** Awareness, Knowledge and Belief Regarding Bitter Leaf Use: A Cross-sectional Study in Nigeria

**Name and affiliation researcher:** This study is being conducted by Dr. Obi Peter Adigwe of the National Institute for Pharmaceutical Research and Development.

**Purpose of research:** This study aims at assessing knowledge, belief, attitude and practice on *Vernonia amygdalina* (Bitter leaf) use amongst Nigerians.

**Objectives**

1. To determine if knowledge of nutritional or medicinal value will influence the consumption of *Vernonia amygdalina.*
2. To assess predictors of attitudes towards bitter leaf
3. To determine factors associated with belief towards *Vernonia amygdalina.*
4. To determine if availability will influence the consumption of *Vernonia amygdalina*.
5. To determine the rate of consumption of *Vernonia amygdalina.*

**Study Procedure:** If you are willing to participate in this study, a questionnaire shall be administered to you. You are expected to go through the questions and answer them to the best of your ability. A total number of 400 participants would be required.

**Expected duration of research and of participant(s)’ involvement:** This study would be undertaken within a period of 6 months. You are however required to complete the questionnaire once which is expected to take not more than 7 minutes of your time.

**Risk:** There is no potential risk for participating in this study. However, you are free to quit if you feel there is any potential risk you might have envisaged.

**Benefit:** This outcome of this study can guide government in policy direction towards use of medicinal plants.

**Confidentiality:** Absolute confidentiality will be maintained, all information provided will be used for the purpose of this study only.

**Voluntariness:** Your participation in this research is entirely voluntary.

**Any apparent or potential conflict of interest:** There are no known conflict of interests.

**Statement of person obtaining informed consent:**

I have fully explained this research to ____________________________________ and have given sufficient information, including about risks and benefits, to make an informed decision.

DATE: _____________________ SIGNATURE: _______________________________

NAME: ______________________________________________

**Statement of person giving consent:**

I have read the description of the research or have had it translated into language I understand. I understand that my participation is voluntary. I know enough about the purpose, methods, risks and benefits of the research study to judge that I want to take part in it. I understand that I may freely stop being part of this study at any time. I have received a copy of this consent form and additional information sheet to keep for myself.

DATE: ___________________ SIGNATURE: _________________________________

NAME: _____________________________________________

**Detailed contact information of researcher and NHREC:** This research has been approved by the National Institute for Pharmaceutical Research and Development, Health Research Ethics Committee and can be contacted at Plot 942 Cadastral Zone C16, Idu Industrial District 1B, P.M.B 21 Garki, Abuja. Email is niprdhrec@gmail.com. In addition, if you have any question about your participation in this research, you can contact principal investigator, Dr. Obi Peter Adigwe at National Institute for Pharmaceutical Research and Development, Idu Industrial Area, P.M.B. 21 Garki, Abuja, Nigeria.

PLEASE KEEP A COPY OF THE SIGNED INFORMED CONSENT
